# A Glucose Fuel Cell for Implantable Brain–Machine Interfaces

**DOI:** 10.1371/journal.pone.0038436

**Published:** 2012-06-12

**Authors:** Benjamin I. Rapoport, Jakub T. Kedzierski, Rahul Sarpeshkar

**Affiliations:** 1 Department of Electrical Engineering and Computer Science, Massachusetts Institute of Technology, Cambridge, Massachusetts, United States of America; 2 Advanced Silicon Technology Group, Lincoln Laboratory, Massachusetts Institute of Technology, Lexington, Massachusetts, United States of America; 3 M.D.– Ph.D. Program, Harvard Medical School, Boston, Massachusetts, United States of America; Université de Technologie de Compiägne, France

## Abstract

We have developed an implantable fuel cell that generates power through glucose oxidation, producing 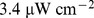 steady-state power and up to 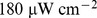 peak power. The fuel cell is manufactured using a novel approach, employing semiconductor fabrication techniques, and is therefore well suited for manufacture together with integrated circuits on a single silicon wafer. Thus, it can help enable implantable microelectronic systems with long-lifetime power sources that harvest energy from their surrounds. The fuel reactions are mediated by robust, solid state catalysts. Glucose is oxidized at the nanostructured surface of an activated platinum anode. Oxygen is reduced to water at the surface of a self-assembled network of single-walled carbon nanotubes, embedded in a Nafion film that forms the cathode and is exposed to the biological environment. The catalytic electrodes are separated by a Nafion membrane. The availability of fuel cell reactants, oxygen and glucose, only as a mixture in the physiologic environment, has traditionally posed a design challenge: Net current production requires oxidation and reduction to occur separately and selectively at the anode and cathode, respectively, to prevent electrochemical short circuits. Our fuel cell is configured in a half-open geometry that shields the anode while exposing the cathode, resulting in an oxygen gradient that strongly favors oxygen reduction at the cathode. Glucose reaches the shielded anode by diffusing through the nanotube mesh, which does not catalyze glucose oxidation, and the Nafion layers, which are permeable to small neutral and cationic species. We demonstrate computationally that the natural recirculation of cerebrospinal fluid around the human brain theoretically permits glucose energy harvesting at a rate on the order of at least 1 mW with no adverse physiologic effects. Low-power brain–machine interfaces can thus potentially benefit from having their implanted units powered or recharged by glucose fuel cells.

## Introduction

As implantable electronic devices become increasingly prevalent in the diagnosis, management, and treatment of human disease, there is a correspondingly increasing demand for devices with unlimited functional lifetimes that integrate seamlessly into their host biological systems. Consequently, a holy grail of bioelectronics is to engineer biologically implantable systems that can be embedded without disturbing their local environments while harvesting from their surroundings all of the power they require. In particular, micropower implantable electronics beg the question of whether such electronics can be powered from their surrounding tissues. Here we discuss how to construct an implantable glucose fuel cell suitable for such applications, and how it may potentially be powered from cerebrospinal fluid in the brain.

### Bioimplantable Power Sources

Various solutions to the problem of providing power to biologically implanted devices have been proposed, prototyped, or implemented. Two principal solutions are currently in widespread use: single-use batteries, such as those used in implantable pulse generators for cardiac pacing, defibrillation, and deep brain stimulation, which are designed to have finite lifetimes and to be replaced surgically at intervals of several years [1]; and inductive power transfer, typically accomplished transcutaneously at radio frequencies, as in cochlear implants [2], [3]. Inductive schemes can be used either to supply power continuously or to recharge an implanted power source. Recent advances in battery technology and related fields, leading to increased energy and power densities in small devices such as supercapacitors [4], [5] as well as thin film lithium and thin film lithium ion batteries [6], will facilitate improvements in systems based on these two solutions, particularly by shrinking battery sizes and extending battery lifetimes.

### Power Scavenging and Power Requirements for Implantable Electronics

Systems for transducing light [7], heat [8], mechanical vibration [9], as well as near- [3], [10] or far-field [11] electromagnetic radiation, into electrical energy, have been described and implemented. Several of these energy-harvesting techniques, as well as electronic design techniques required to make use of them, have been discussed in depth in [12], [13]. Here we focus on powering biologically implanted devices by harvesting energy from glucose in the biological environment.

The emergence of ultra-low-power bioelectronics as a field over the last decade [13] has led to the development of highly energy-efficient, implantable medical devices with power budgets in the microwatt regime. This new generation of low-power devices has driven interest in a range of sustainable power sources and energy scavenging systems that, while impractical for conventionally designed electronic devices, are entirely practical in the context of micropower electronics [13]. For example, in brain– machine interfaces, the combination of low-power circuit design [14] and adaptive power biasing [15] can be used to build sub-microwatt neural amplifiers for multi-electrode arrays. Impedance-modulation radio-frequency (RF) telemetry techniques can drastically reduce implanted-unit power consumption and operate at less than 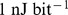 even for transcutaneous data rates as high as 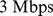 in brain– machine interfaces. Finally, ultra-low-power analog processing techniques [13], [16] can enable 100-channel neural decoding at micropower levels [17], [18] and dramatically reduce the data rates needed for communication, further reducing total power consumption. The combination of these advances in energy-efficient amplification, communication, and computation implies that implanted components in brain– machine interfaces that operate with tens of microwatts of total power consumption are feasible today. Therefore, implantable biofuel cells such as the one presented here, which generate power at densities on the order of 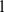
–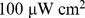, provide useful power sources for such ultra-low-power implantable medical devices.

### Glucose Fuel Cells

A fuel cell generates power by catalyzing complementary electrochemical reactions (oxidation and reduction) at a pair of corresponding electrodes (the anode and cathode, respectively), as a reducing-agent fuel flows across the anode and an oxidant flows across the cathode. The fuel substrate is electrooxidized at the anode, which collects the liberated electrons and conducts them through an external load to the cathode. Typically an ion-selective membrane partitions the anode and cathode into separate chambers, facilitating a unidirectional flow of protons, generated via oxidation at the anode, to the cathode. The protons arriving at the cathode through the solution, the electrons arriving at the cathode through the external circuit, and the oxidant at the cathode undergo a redox reaction that reestablishes charge neutrality in the overall cell.

One approach to harvesting energy from a physiologic environment is to extract it from physiologically available biological fuel substrates such as glucose. In a glucose-based biofuel cell, glucose is oxidized at the anode, while oxygen is reduced to water at the cathode. The nature of the catalyst residing at the anode determines the extent of glucose oxidation and the associated oxidation products. Three major design paradigms for glucose-based fuel cells have been described, differing principally in the materials used to catalyze electrode reactions: In one paradigm the catalysts are abiotic, solid-state materials; in the second paradigm the catalysts are isolated enzymes fixed to electrode substrates; and in the third paradigm oxidation is performed by exoelectrogenic bacterial biofilms colonizing a fuel cell anode. Numerous designs representing each of these fuel cell paradigms have been described and reviewed in the scientific and patent literatures. An extensive review of the scientific and patent literatures on abiotic implantable glucose fuel cells is provided in [19]. Bioimplantable fuel cells based on enzymatic catalysis are reviewed in [20]. Microbial fuel cells are reviewed in [21].

These three principal catalytic schemes yield systems that differ markedly from one another in efficiency and robustness.

Enzyme-based glucose fuel cells have high catalytic efficiency, which together with their small size results in high volumetric power density, yielding up to 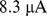, and 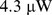 of total power, in systems with footprints on the order of 1 mm^2^ and volumes less than 10^–2^ mm^3^ [20]. Such fuel cells are often constructed as tethered-enzyme systems, in which oxidation and reduction of fuel cell substrates are catalyzed *ex vivo* by enzymes molecularly wired to threads of conductive material. Enzyme-based glucose fuel cells described in the recent literature have typically generated on the order of 100 µW cm^−2^ [22], [23]. Fuel cells of this kind may be capable of continuous operation for up to several weeks, but their lifetimes are limited by the tendency of enzymes to degrade and ultimately degenerate with time.

Using living microorganisms, such as exoelectrogenic bacteria, to catalyze the anodic reaction results in complete oxidation of glucose, liberating twenty-four electrons per molecule of glucose consumed. Microbial fuel cells are thus very efficient catalytically, and can produce more than 1300 µA cm^−2^ and 1900 µW cm^−2^ [24], [25]. Moreover, in contrast with enzymatic systems, which have short lifetimes limited by the degradation of tethered enzymes *ex vivo*, microbial fuel cells are inherently self-regenerating: they use a fraction of input biomass to power and supply molecular substrates for maintenance functions such as resynthesis of degraded enzymes [21]. Microbial glucose fuel cells described in the recent literature have typically generated on the order of 1000 µW cm^−2^ [22], [23], [26]. However, microbial fuel cells of the present generation are not yet suitable for biologically implanted applications. The prospect of implanting even nonpathogenic bacteria raises concerns of safety and biocompatibility.

Solid-state anode catalysts such as those we use in the work described here are capable of oxidizing glucose to gluconic acid, liberating one pair of electrons, and yielding further oxidation products with reduced probability [19], [27]. As a result, they represent the least catalytically efficient of the three design paradigms we consider. Yet while glucose fuel cells based on solid-state catalysts typically only generate on the order of 1–10 µA cm^−2^ and 1–10 µW cm^−2^ [19], they have proven reliable as implantable power sources for many months [28], [29].

### Cerebrospinal Fluid as a Physiologic Niche Environment for a Fuel Cell

An innovation in the work described here is the use of cerebrospinal fluid as a physiologic niche for an implantable power source. Implantable fuel cells have typically been designed for use in blood or interstitial fluid; to the best of our knowledge, the operation of a biofuel cell in the cerebrospinal fluid has not previously been described (we are aware of one incidental reference, made in [30]). The cerebrospinal fluid represents a promising environment for an implantable fuel cell: It is virtually acellular, it is under minimal immune surveillance, it has a hundred-fold lower protein content than blood and other tissues and is therefore less prone to induce biofouling of implanted devices, and its glucose levels are comparable to those of blood and other tissues [31]. The bioavailability of glucose and oxygen to a fuel cell residing in the subarachnoid space is addressed in detail in the Methods subsection entitled ‘Brain and Cerebrospinal Fluid as Sites for an Energy-Harvesting Fuel Cell.’

### Fuel Cell Design

**Figure 1:**
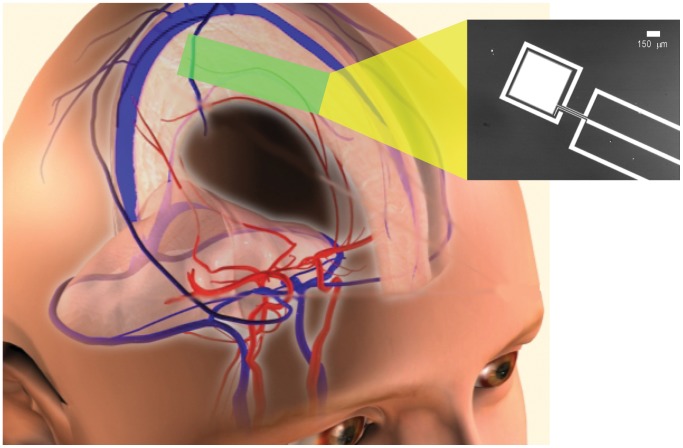
Power Extraction from Cerebrospinal Fluid by an Implantable Glucose Fuel Cell. Conceptual schematic design for a system that harvests power from the cerebrospinal fluid, showing a plausible site of implantation within the subarachnoid space. The inset at right is a micrograph of one prototype, showing the metal layers of the anode (central electrode) and cathode contact (outer ring) patterned on a silicon wafer. *Image Credit: Meninges and Vascular Anatomy courtesy of the Central Nervous System Visual Perspectives Project, Karolinska Institutet and Stanford University.*

**Figure 2:**
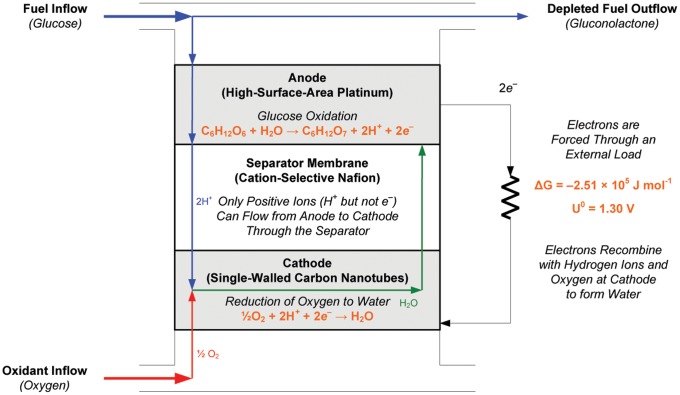
General Operational Scheme for an Implantable Glucose Fuel Cell. This schematic conceptually illustrates the structure of an abiotically catalyzed glucose fuel cell, including the essential half-cell and overall reactions, the sites at which they occur within the system, and the flows of reactants and products.

**Figure 3:**
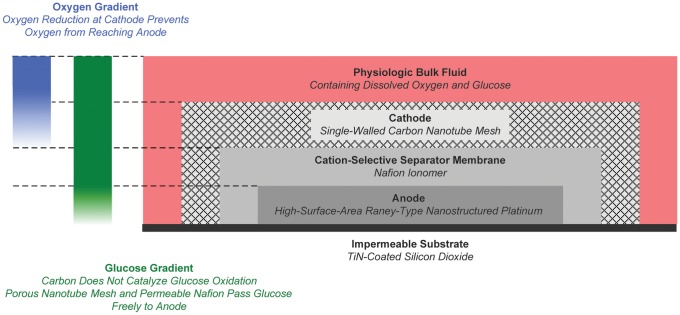
Glucose Fuel Cell in Cross Section. This schematic cross-section of the glucose fuel cell illustrates the structure of the device, as well as the oxygen and glucose concentration gradients crucially associated with its cathode and anode half-cell reactions, and underlying their respective site specificity.

A variety of mechanical designs for bioimplantable biofuel cells have been described in a literature spanning at least half a century [19]. A number of physical, electronic, and electrochemical factors also influence the voltage and current output of a biofuel cell. These include fuel cell and electrode geometry; electrode and membrane spacings; redox potentials of fuel cell components; internal and load impedances; and environmental conditions in which the cell operates, including fuel substrate concentration, environmental temperature and pH, and the presence of chemical species capable of driving parasitic side reactions. Many of these aspects of fuel cell design have been modeled in detail and measured empirically in real systems [32], [33]. As described in the Methods Section and as shown in Figures 1, 2, and 3, the novel design and manufacturing process we describe here is a version of a classic half-open, two-chamber design [19], [34], [35], sized and shaped to fit a particular anatomic compartment, and constructed using semiconductor fabrication techniques that could in principle permit manufacture together with integrated circuits on a single silicon wafer.

### Structure of This Paper

This paper is structured as follows. In the Results and Discussion Section we describe the performance of our glucose fuel cell. In the Methods Section we address several topics in detail. First, we discuss solid-state catalysis of glucose oxidation from a theoretical perspective, and explain the operating principles of our fuel cell, including its mechanism of separating the oxidation and reduction reactions, even though their reactants, glucose and oxygen, naturally occur mixed in physiologic compartments. Next, we describe our CMOS-compatible process for fabricating implantable glucose fuel cells. In that subsection we also describe our approach to characterizing the materials and electrochemical properties, as well as the power-generating performance of the fuel cells. We then discuss the power available from circulating glucose in human physiologic compartments, and the suitability of the cerebrospinal fluid as a physiologic niche for power harvesting. We describe a detailed model of glucose and oxygen consumption by a fuel cell implanted in the subarachnoid space surrounding the human brain, and analyze the impact of such a fuel cell on glucose and oxygen homeostasis.

## Results and Discussion

### Device Characterization

#### Anode surface roughness by scanning electron microscopy

**Figure 4:**
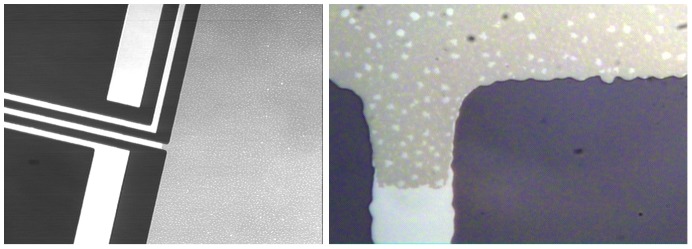
Anode Roughening. These optical micrographs illustrate the effect of the roughening technique on the anode surface, showing the atomically smooth platinum traces (narrow metallic strips) leading to the anode in contrast with the anode itself (large rectangular area). The roughness of the anode surface is detectable optically as an abrupt change in color and texture. The image at right is an enlargement of the central region of the image at left, focusing on the boundary between the smooth and rough platinum surfaces (rotated with the wire trace set vertical). Scale: The wire traces (left and bottom left, respectively) are 100 *µ*m wide.

**Figure 5:**
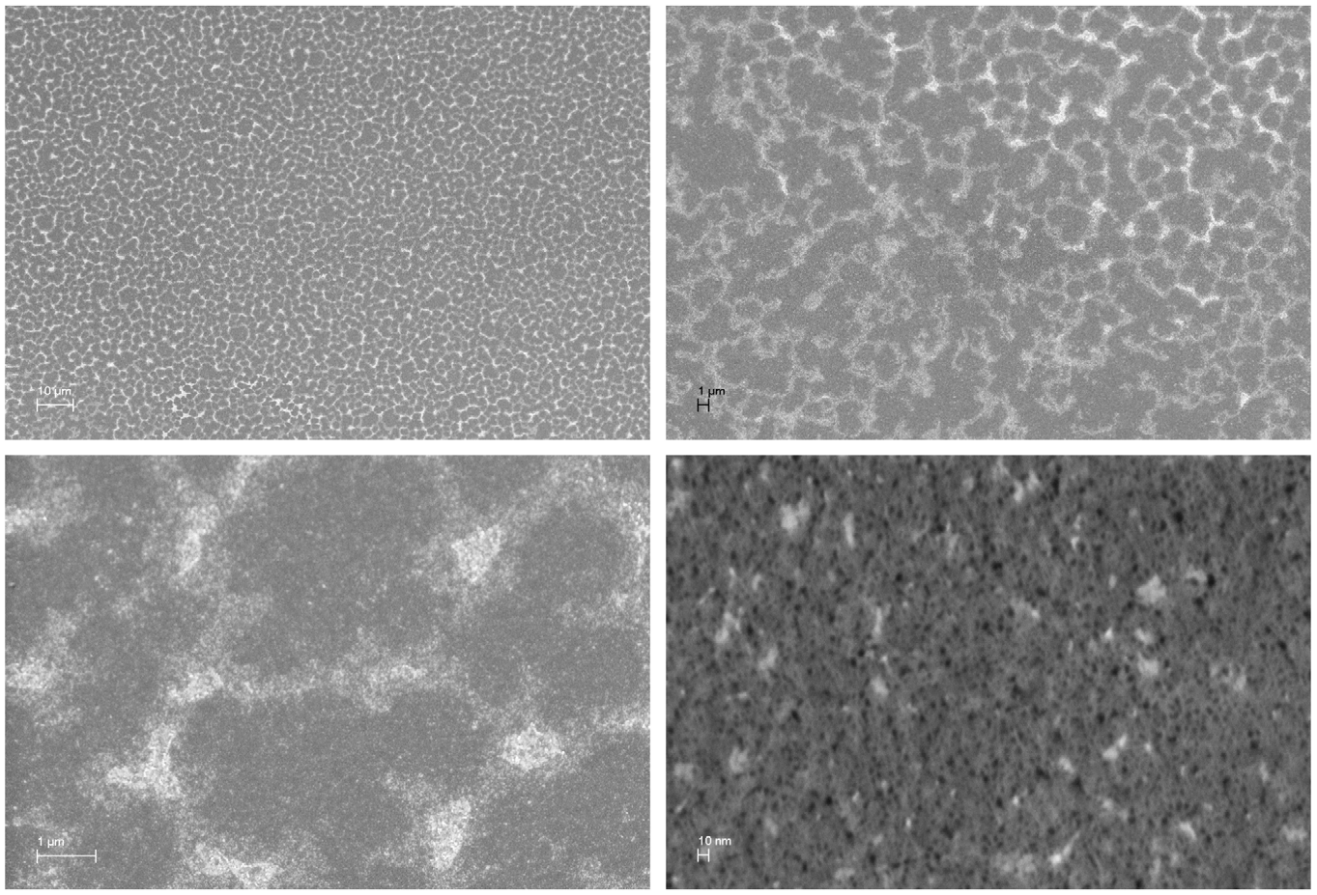
Anode Micro- and Nanostructure. This set of scanning electron micrographs, taken of a fuel cell anode at increasing levels of magnification (as indicated by the scale bars in each image), illustrates the effects of the roughening procedure on electrode surface structure over a hierarchy of length scales from nanometers to micrometers.

**Figure 6:**
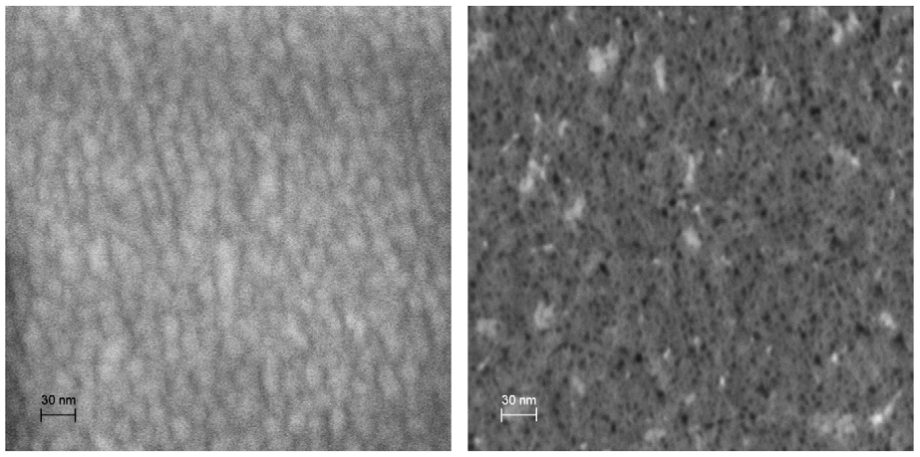
Nanostructural Effects of Surface Roughening. This pair of scanning electron micrographs, taken at the same level of magnification, illustrates the effects of our surface roughening technique. The image at left is a high-magnification image of atomically smooth platinum deposited by evaporation on silicon dioxide. The image at right is taken from one of our roughened anodes, and shows the highly porous nanostructure of the electrode.

The efficiency of the fuel cell critically depends on its ability to catalyze the oxidation of glucose at the anode. Our device uses a solid-state platinum anode catalyst, whose catalytic capacity is directly related to the number of atomic sites it can provide on its surface. We describe a CMOS-compatible process for electrode surface roughening to increase effective electrode surface area, and hence catalytic capacity, in the Methods Section. Briefly, by alloying the platinum electrode with aluminum, and then reactively etching away all of the aluminum, we generate a high-surface-area anode with a nanostructure very different from that of atomically smooth platinum. Here, we illustrate the effects of that roughening procedure in a series of micrographs. Figure 4 shows the contrast between atomically smooth and roughened platinum metal layers as patterned on a silicon dioxide wafer substrate, as seen under optical microscopy. Figure 5 shows a series of scanning electron micrographs, taken at increasing magnifications (from single-micrometer to single-nanometer resolution), and showing the persistence of pore-like structures at every scale, as expected on removing one element of a bimetallic alloy. The scanning electron micrographs in Figure 6 demonstrates the contrast between atomically smooth platinum and roughened platinum at the nanometer scale.

#### Anode surface roughness by atomic force microscopy

**Figure 7:**
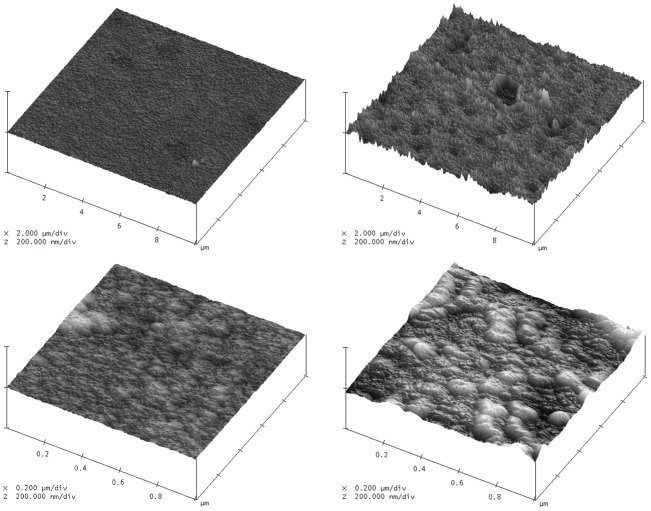
Atomic Force Microscopic Measurements of Anode Surface Roughness. Atomic force microscopic measurements of *z*-direction (plane-normal) surface roughness at the 10 µm and 1 µm scales (upper and lower images, respectively), comparing atomically smooth platinum (left images) with the roughened anodes we describe here (right images).

We used atomic force microscopy to quantify the surface area enhancements generated by the anode roughening procedure we employed, using a high-aspect-ratio silicon nitride probe with radius of curvature 5 nm. Figure 7 shows two pairs of surface scans, contrasting atomically smooth platinum with roughened platinum at 10 µm and 1 µm resolution. While this technique is limited both by the geometry of the scanning probe and by the single-axis nature of the measurements, variance in surface height nevertheless provides a quantitative means of assessing surface roughness. Sampling at 256 sites over 10–µm -square patches, we measured a variance of 10.3 nm in the roughened anodes, as compared with a variance of 1.2 nm in atomically smooth platinum (an 8.5-fold increase in vertical-, or z-roughness).

#### Biocompatibility

As we note elsewhere, the cerebrospinal fluid represents a promising niche for an implantable fuel cell in that it is virtually acellular and has a hundred-fold lower protein content than blood and other tissues, so is therefore less prone to induce biofouling of implanted devices; in addition, it is relatively free from immune surveillance, and its glucose levels are comparable to those of blood and other tissues [31].

Furthermore, Nafion is biocompatible [36], and in using it as an outermost layer encapsulating our fuel cell we enhance the biocompatibility of the entire device. Moreover, as a cation-selective ionomer, Nafion is impermeable to negatively charged proteins, small molecules, and ions, that are present in the physiologic environment and are known to cause fouling of catalytic electrodes. Nafion encapsulation has been used in the context of glucose sensors to increase the functional lifetimes of implanted electrodes [37].

While the designs described here are intended to optimize biocompatibility, we note that in evaluating these designs, biocompatibility must ultimately be assessed in the challenging context of *in vivo* testing.

### Power Output and Electrochemical Characterization

**Figure 8:**
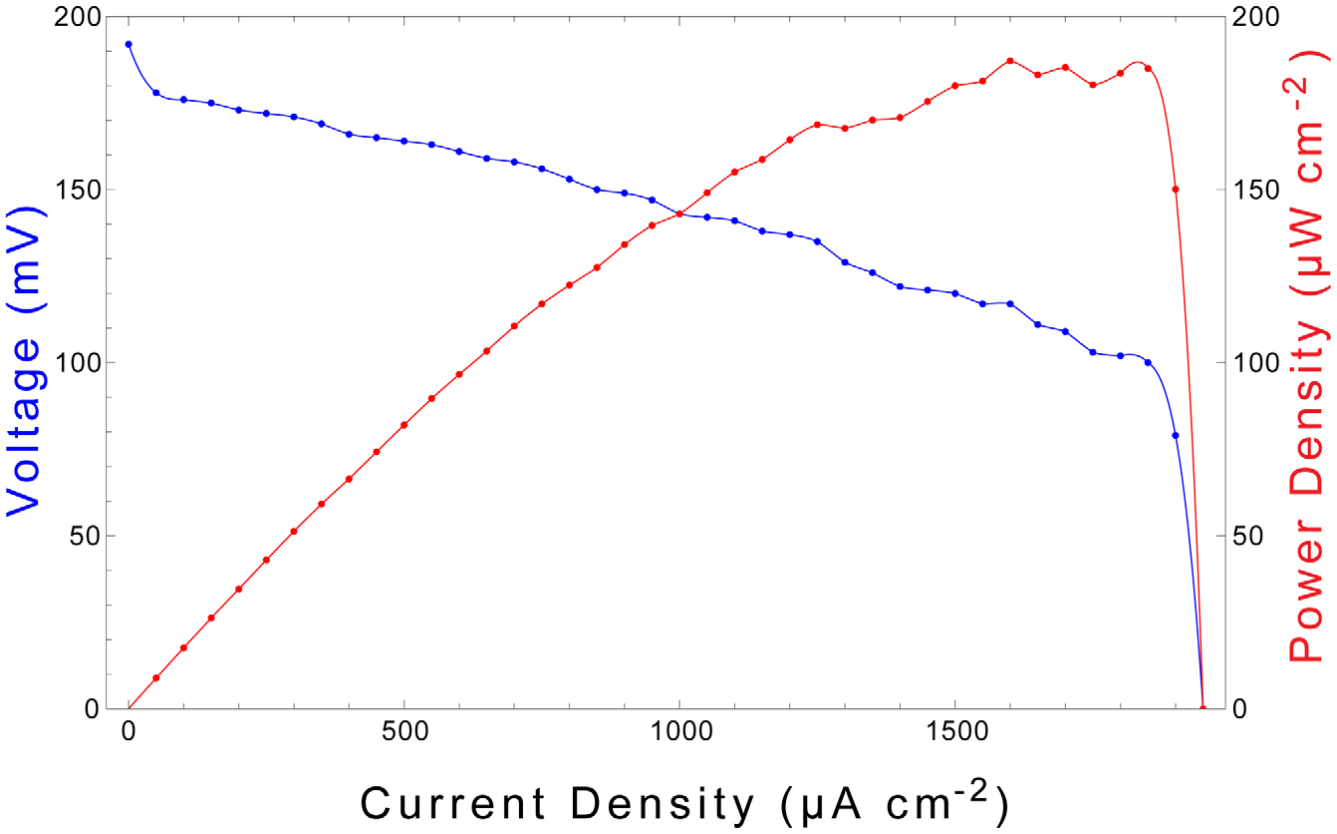
Fuel Cell Polarization Curve. The performance of the fuel cell is characterized through its output voltage (blue, left axis) and power density (red, right axis) as functions of output current density. A 2 mm^2^ device exhibits an open-cell voltage of 192 mV and achieves maximum power output of more than 180 µW cm^−2^ when sourcing 1.5–1.85 mA cm^−2^.

**Figure 9:**
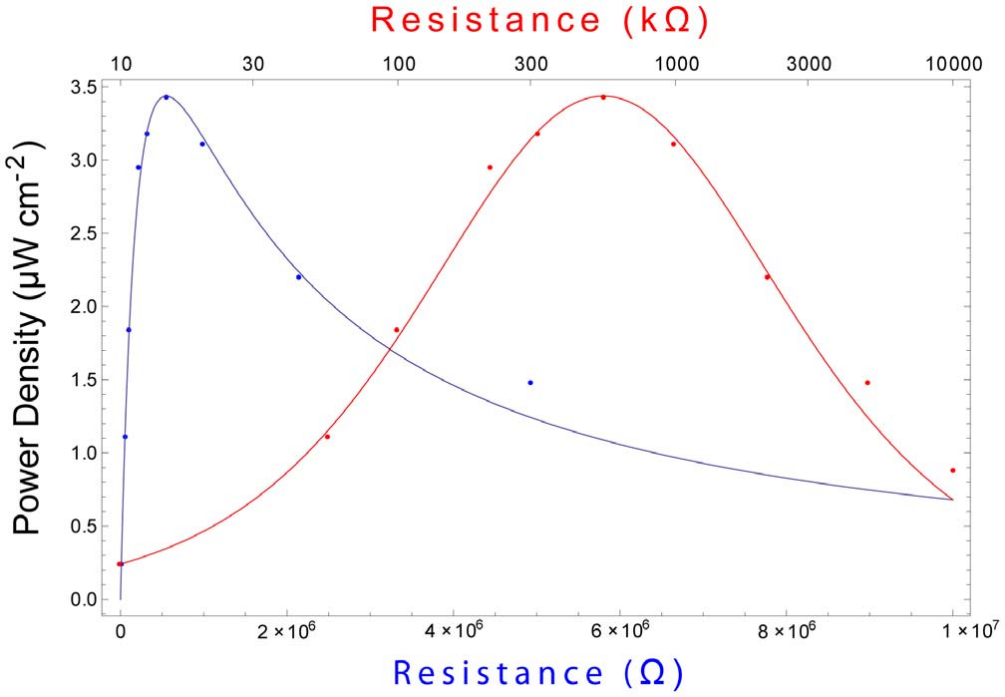
Impedance Matching to Maximize Output Power. Steady-state output power of the fuel cell exhibits characteristic second-order dependence on the magnitude of the resistive load. A 1 mm^2^ device achieves maximum steady-state power output of 3.4 µW cm^−2^ when driving a load of 550 kΩ. (Blue curve, lower horizontal axis, linear scale; red curve, upper horizontal axis, logarithmic scale.).

**Figure 10:**
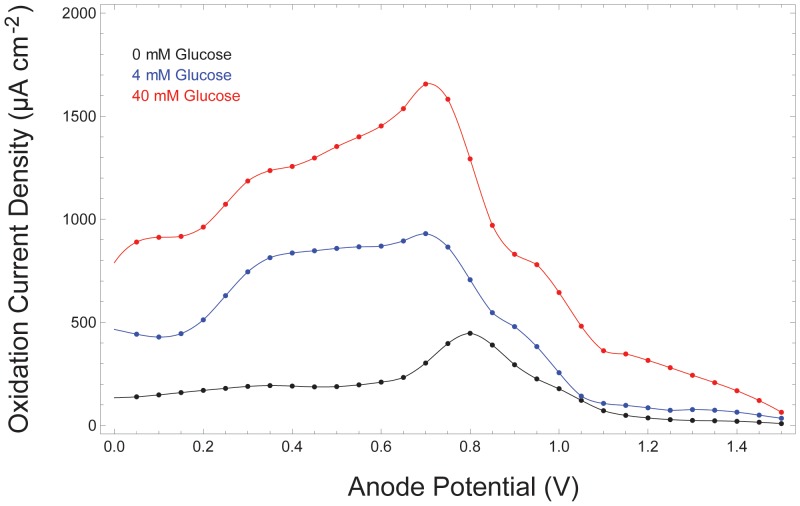
Oxidation Current Depends on Glucose Concentration. The oxidation current generated by the fuel cell anode, characterized here via cyclic voltammetry, varies with glucose concentration.

Equation 3 indicates that our system liberates 2 electrons per glucose molecule oxidized. In the fuel cell system, the reactions described in Equation 3 occur simultaneously and in parallel at many atomic, catalytic sites distributed across the surface of the anode. Fuel cell performance is therefore determined not only by the numeric efficiency of the oxidation reactions described in Equation 3, but also by the number of catalytic sites available at the anode (essentially determined by anode surface area) and by the rate at which the reactions occur. One measure of fuel cell performance is therefore the rate at which oxidation electrons are collected (and driven through an external load), normalized to anode surface area. Hence, it is customary to measure fuel cell current (and power) area densities, and we characterize the performance of our devices in these terms in Figures 8, 9, and 10.

#### Power output

We first characterize the glucose fuel cell through a polarization curve, Figure 8, which shows the cell potential (and corresponding output power density) maintained while sourcing current at maximum transient rates. Using the fuel cell as a current source by operating a potentiostat in controlled-current mode, we drew current from the fuel cell at a rate increasing from zero by 10 µA cm^−2^ s^−2^, until the cell potential was abolished. Figure 8 shows the cell voltage and power output generated during this procedure. The fuel cell generated an open-circuit voltage of 192 mV, and transiently generated peak power of more than 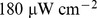 when sourcing 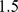
–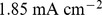. Deep discharges at currents greater than 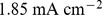 for longer than 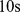 were found to damage the fuel cells irreversibly.

Techniques that exploit resonant transformer action can be used to efficiently convert even 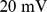 energy-harvesting outputs to 
–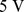 levels needed for powering electronic chips [38]. In an actual brain– machine interface, such techniques will be needed to increase the voltage level of our glucose fuel cell as well.

#### Impedance matching for optimizing power output

We determined the steady-state power output of the glucose fuel cell when driving a range of resistive loads. The results are shown in Figure 9 for a 1 mm^2^ device, which achieved maximum steady-state power output of 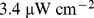 when driving a load of 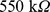. The curves shown with the data in Figure 9 represent the output power density function
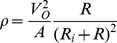
(1)where 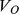 denotes the open-circuit voltage, 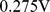; A denotes the anode area, 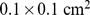; 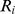 denotes the inferred internal resistance of the cell, 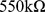; *R* denotes the variable load resistance; and 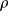 denotes the output power density.

#### Power output as a function of glucose concentration

We tested our fuel cells in standard 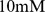 phosphate buffered saline (containing 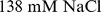, 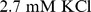, 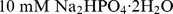, and 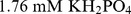) at 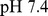 to simulate the physiologic environment of the cerebrospinal fluid, loading the medium with glucose at various concentrations. The performance data provided earlier in this section was obtained in the context of 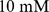 glucose [39]. During testing we measured but did not regulate system temperature, which ranged from 18°C to 24°C.

As expected, and as quantified in Figure 10, the anodic current generated by the fuel cell varies as a function of ambient glucose concentration. Figure 10 shows the anodic current from a fuel cell anode, derived through cyclic voltammetry, swept through oxidizing potentials at 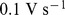 while the anode was kept in media containing glucose at concentrations from 0 to 40 mM. Although physiologic concentrations are of primary interest in the applications we discuss here, we have observed that oxidation current continues to increase as a function of glucose concentration well beyond the upper limit of the normal physiologic range in cerebrospinal fluid, approximately 4.4 mM. Figure 10 also illustrates, in accord with the observations of others [40], that concentration-dependent increases in oxidation current (reflected by the areas of the glucose oxidation peaks in the region of 300 to 700 mV) are sublinear: oxidation current rises only slowly in response to order-of-magnitude increases in glucose concentration. The sublinearity of this relationship confirms that fuel cell performance is not primarily substrate-limited at or near physiologic glucose concentrations.

## Methods

### Solid-State Catalysis of Glucose Oxidation

The electrochemical reaction mechanisms of direct glucose fuel cells are discussed in detail by Kerzenmacher and colleagues in their thorough review of energy harvesting by implantable, abiotically catalyzed glucose fuel cells [19]. The complete oxidation of glucose to carbon dioxide and water is associated with the transfer of 24 electrons per molecule of glucose, as described by the reactions in Equation 2:
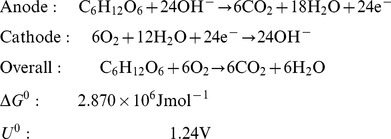
(2)


However, studies have confirmed that glucose is not completely oxidized when its oxidation is mediated by traditional, solid state catalysts; the theoretical maximum rate of electron transfer from glucose oxidation is not achieved in abiotically catalyzed glucose fuel cells. Instead, glucose is principally oxidized to gluconolactone in the reaction described by Equation 3, which transfers only a single pair of electrons:
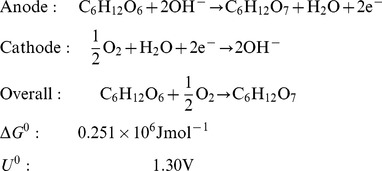
(3)


Gluconolactone is typically then hydrolyzed to form gluconic acid. In principle, solid-state catalysts are capable of oxidizing gluconolactone further, and high-pressure liquid chromatography has detected tartaric and oxalic acids in such systems (resulting from 14- and 22-electron-transfer processes, respectively), among other oxidation products. In practice, the mean number of electrons transferred per molecule of glucose oxidized depends on the nature of the catalyst and on thermodynamic properties of the system, such as ambient pH. Gebhardt and colleagues have shown that the mean number of electrons transferred per molecule of glucose by Raney-type catalysts can be up to 17 [27]. As this family of high-surface-area catalysts has also been shown to generate current densities an order of magnitude greater than smooth noble metal catalysts, we chose to use Raney platinum anodes to catalyze glucose oxidation in our fuel cells.

### Separation of Anode and Cathode Reactions

Maintenance of a net potential difference between the anode and the cathode of a fuel cell requires separation of the oxidation and reduction half-reactions in a way that restricts each to only one of the fuel cell electrodes. As discussed in [19] and elsewhere, biologically implantable fuel cells pose a particular design challenge in that the fuel (glucose) and the oxidant (oxygen) must be extracted from same physiologic fluid, in which both are dissolved. By contrast, fuel cells are traditionally configured so as to isolate the anode from the cathode fluidically, in a compartmental arrangement that separates the two half-cell reactions.

The traditional configuration permits delivery of fuel to the anode and oxidant to the cathode via separate fluidic channels, an arrangement that can be effective because it physically prevents reduction (typically of oxygen) from occurring at the anode, and prevents oxidation of the fuel substrate from occurring at the cathode. These reverse reactions cause electrochemical short circuits by allowing both oxidation and reduction to occur at each electrode, eliminating the net potential difference across the fuel cell electrodes.

Preventing electrochemical short circuits in an implantable glucose fuel cell requires a different approach to restricting the fuel oxidation and oxygen reduction to the anode and cathode, respectively. Several designs are reviewed in [19]. We have adopted a modified version of a design first proposed by von Sturm and colleagues [34], [35], which uses an oxygen-selective cathode catalyst to shield a nonselective anode from oxygen, while allowing glucose to reach the anode without reacting at the cathode. This scheme is illustrated in Figure 3.

As indicated in Figure 3, our fuel cell has a laminar structure: a porous mesh of single-walled carbon nanotubes, embedded in Nafion, comprises the cathode and forms the outermost layer; this layer is followed (from the outside in) by a Nafion separator membrane; a roughened (Raney-type) platinum anode; and finally the impermeable, silicon dioxide substrate. In this configuration, oxygen from the physiologic environment reacts at the carbon cathode, resulting in a gradient in the oxygen concentration that reaches its minimum, near zero, at the surface of the anode [35], [40]. The near absence of oxygen at the anode surface minimizes the rate of oxygen reduction at the anode, which in turn minimizes the electrochemical short-circuiting effects of such reactions. On the other hand, because carbon does not catalyze glucose oxidation, and because Nafion is permeable to glucose, physiologic glucose passes through the pores in the nanotube mesh unimpeded.

### Microfabrication Methods

In contrast with most previous work in the area of glucose fuel cells [40], and in fulfillment of a recognized requirement for advancement of the field [41], [42], we manufactured our fuel cells entirely within a Class 10 (ISO 4) cleanroom, using microfabrication techniques and processing standards that are completely compatible with contemporary CMOS (complementary metaloxidesemiconductor) integrated circuit manufacturing protocols. As a result, the fabrication methods we describe here can be used to enable wafer-level integration of fuel cell power sources with electronic circuits and microfluidics, facilitating the development of completely self-sufficient, embedded electronic and microfluidic systems.

#### Substrates, masks, and lithography

Electrodes, separator membranes, wire traces, and metal contacts were all patterned using conventional photolithography, with transparency masks printed to 2 µm feature-size tolerance (Infinite Graphics, Minneapolis, Minnesota). As substrates, we used 500 nm surface layers of silicon dioxide on conventional, 150-millimeter-diameter (6-inch) silicon wafers. To promote platinum adhesion, we deposited a 2 nm coat of titanium nitride on the oxide by evaporation.

#### Raney catalyst anode

**Figure 11:**
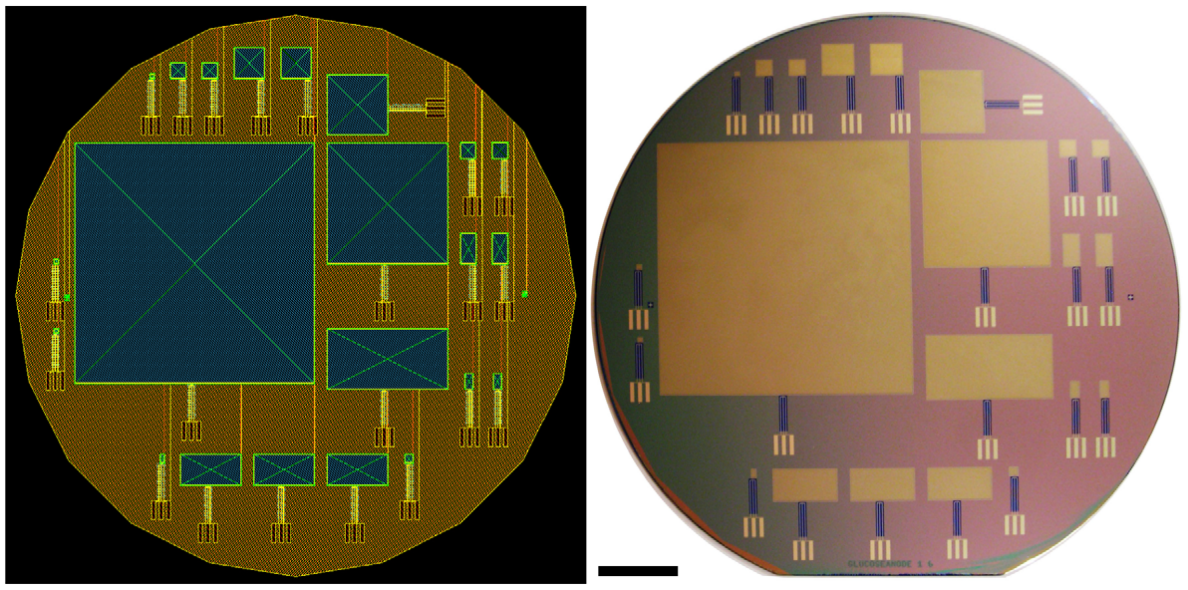
Photolithography Masks and Fabricated Fuel Cells. The image at left shows a set of superimposed photolithographic masks for glucose fuel cells of various sizes, arranged for fabrication on a silicon wafer 150 mm (6 inches) in diameter. The largest device depicted has an anode that measures 64 mm by 64 mm. The anodes of the other fuel cells shown are scaled-down versions of the large device, with length and width alternately reduced by factors of two. The schematic was constructed by overlaying the four process layers: yellow, platinum; orange, roughened platinum anode (aluminum deposition for annealing); blue, Nafion; green, cathode (single-walled carbon nanotubes in Nafion). The photograph at right shows the corresponding silicon wafer as fabricated. Scale Bar: 2 cm.

We fabricated Raney-type, activated platinum catalytic anodes using an approach similar to those described by several other groups [27], [43], [44]. This technique increases the catalytic capacity of a platinum electrode through roughening, converting an atomically smooth layer of platinum into a high-surface-area electrode. The overall approach involves patterning a platinum-aluminum alloy, then etching the aluminum out of the alloy to leave behind extremely porous platinum with a nanostructure similar to that described by Attard and colleagues [45], [46]. Our implementation proceeded as follows. We patterned all metal structures using a single mask, including the anode, cathode contact ring, wire traces, and electrical contacts, as in Figure 11, depositing 100 nm of platinum by evaporation. We then deposited 100 nm of aluminum over the entire surface of the wafer. Using a second mask, we patterned photoresist over the regions designated for the anodes, protecting them from the etchant acting in the following step: using tetramethylammonium hydroxide (TMAH), we etched away the aluminum in all areas except for those designated as anodes. After stripping the remaining resist, we annealed the platinum and aluminum layers at 300°C for 60 minutes. This anneal step generated a platinum-aluminum alloy in the regions designated for the anodes. We then repeated the aluminum etch step, this time without protecting the anodes, in order to remove the aluminum from the alloy formed in the anode regions. This second etch produces high-surface-area Raney-type catalytic anodes, as shown in Figures 4, 5, 6, and 7, and as discussed in the associated Methods subsections.

#### Nafion separator membrane and biocompatible encapsulation layer

Several groups have reported difficulty incorporating Nafion, a preferred ion-selective membrane in fuel cell applications, into microfabrication processes. Patterning Nafion lithographically does present some difficulties, as described by Gold and colleagues [47] and others. Indeed, some groups have recently proposed separation membranes composed of other materials [48]. We used the following liftoff process for depositing Nafion on silicon dioxide and platinum.

We selected the areas to be coated with Nafion by first coating the wafers with 15–20 µm of photoresist (AZ 4620) and using a third mask to pattern and expose the regions to be coated with Nafion. We have found that using such thick layers of resist facilitates proper Nafion patterning and liftoff by enabling patterned regions of Nafion to be isolated in deep resist wells formed at the wafer surface, following resist development and evaporation of the Nafion dispersion solvent.

From a standard Nafion liquid dispersion (Nafion DE 521, DuPont), we formed a 0.83% Nafion solution via 1∶5 dilution in 2-propanol. In a modified version of the protocol described in [49], we used a spin-on process to coat our wafers with the resulting dispersion, spinning the wafers at 750–1000 rpm for 3–10 s. In order to cure the Nafion and facilitate bonding to the substrate [50], we heated the wafers in a convection oven at 120°C for 20 minutes. This process generates Nafion layers approximately 60–420 nm in thickness, as measured by spectroscopic ellipsometry (Filmetrics, San Diego, California).

#### Carbon nanotube cathode

**Figure 12:**
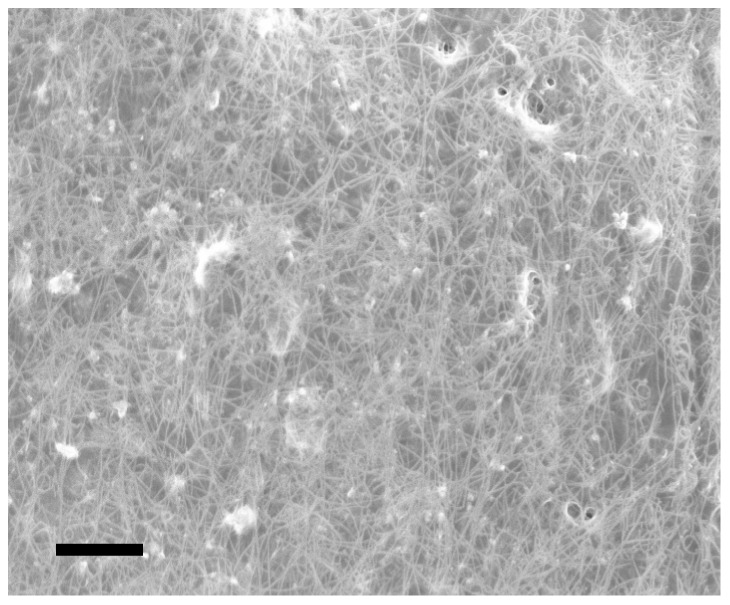
Fuel Cell Cathode. Scanning electron micrograph of the fuel cell cathode, showing the conducting mesh of carbon nanotubes encapsulated in Nafion ionomer. Scale Bar: 1 µm.

The cathode of our fuel cell comprises a conducting mesh of single-walled carbon nanotubes (swCNTs) embedded in Nafion, as shown in Figure 12, in electrical contact with a platinum ring (the cathode contact) on the wafer surface. This design is similar to the one described by Lee and colleagues [51], and other groups have recently exploited nanostructured carbon as a catalytic substrate in glucose fuel cells [52]. The Nafion separator membrane electrically insulates the cathode from the anode, while permitting cationic exchange. Both the separator membrane and the cathode permit ambient glucose to reach the anode surface: Nafion is inherently permeable to glucose and the porosity of the swCNT mesh allows free transport of glucose.

We constructed the Nafion-coated swCNT mesh by allowing it to self-assemble. Using a procedure similar to the one described in [53], we suspended previously synthesized and purified swCNTs (Sigma-Aldrich Corporation) at a concentration of 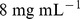 in an 0.83% Nafion dispersion of the kind described earlier in this Methods Section, under ultrasonic agitation for 60 minutes. We then patterned the Nafion-coated swCNT cathode using a fourth lithographic mask, and spin-on and liftoff processes identical to those described earlier in this Methods Section.

### Models of Energy Harvesting from Glucose in the Brain and Cerebrospinal Fluid

In this section we demonstrate that the cerebrospinal fluid is a physiologic niche particularly well suited for an implanted fuel cell, and we model the impact of an implanted glucose fuel cell on its biological environment. In particular, we derive a model used to determine the extent to which glucose and oxygen levels in the cerebrospinal fluid permit energy harvesting without adverse physiologic consequences.

#### Anatomy and composition of the cerebrospinal fluid

The cerebrospinal fluid (CSF) comprises approximately 150 mL of fluid in the subarachnoid and intraventricular spaces, respectively surrounding and filling hollow structures within the human brain and spinal cord. The CSF is primarily produced by modified ependymal cells of the choroid plexus, located within the ventricles of the brain, as an ultrafiltrate of blood plasma whose composition is also regulated through active transport.

Under normal conditions the CSF is acellular. Its normal glucose content of 45–80 mg dL^−1^ (2.5–4.4 mM) [54] is comparable to, albeit systematically approximately 2-fold lower than that of plasma, which is normally regulated within the range of 70–120 mg dL^−1^ (3.9–6.7 mM) [55]. By contrast, normal CSF protein content is 15–45 mg dL^−1^ [56], which is at least 10^2^-fold lower than the normal range for plasma, 6000–8500 mg dL^−1^ [57]. The ionic composition of CSF resembles that of interstitial fluid, and the two fluids are compared in Table 1.

**Table 1:** Ionic Composition of Cerebrospinal Fluid and Interstitial Fluid.

Species	CSF Concentration	ISF Concentration
Na^+^	154 mM	146 mM
K^+^	3.0 mM	4.1 mM
Cl^–^	128 mM	118 mM
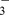 HCO	23 mM	22 mM
H^+^	pH≈7.34	pH≈7.44

Typical values for the principal ionic constituents of mammalian cerebrospinal and interstitial fluids [64].

With regard to the *in vitro* testing conditions for our glucose fuel cell, it is worth noting that a number of formulations for artificial cerebrospinal fluid (aCSF) have been described and developed in efforts to simulate the physiologic fluid (including trace components and gas partial pressures), both for experimental purposes [39] and in order to replace CSF lost during neurosurgical procedures [56]. In clinical practice, however, normal saline is routinely used during neurosurgery to replace CSF.

#### Availability and use of glucose in the cerebrospinal fluid

At physiologic glucose concentrations, high-efficiency biofuel cells such as the microbial biofuel cells developed by Rabaey and colleagues convert glucose to electricity with Coulombic efficiency exceeding 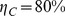 [58], and Lovley and colleagues have reported such efficiencies even at lower glucose concentrations [59]. At the opposite, low-efficiency extreme, abiotically catalyzed biofuel cells typically oxidize glucose incompletely to products such as gluconic acid, yielding only two electrons compared with the theoretical maximum of twenty-four electrons per molecule of glucose [19], corresponding to a maximum Coulombic efficiency of only approximately 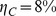. The minimum glucose flux, 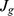, required to fuel a glucose fuel cell generating power *P*, is
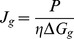
(4)where 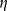 reflects the overall efficiency of the system (the Coulombic efficiency provides an upper bound on the overall efficiency), and 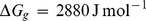 denotes the heat of combustion of glucose. Using Equation 4, we can estimate the minimal glucose flux (amount of glucose per day) required to power a fuel cell generating 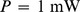:



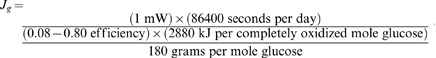
(5)

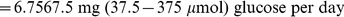
(6)


(The dashes in Equations 5 and 6 are used to denote ranges and should not be mistaken for minus signs.) Since cerebrospinal fluid in a typical adult human is produced at a rate of approximately 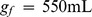 per day [60], the total flow of glucose through the subarachnoid space is approximately 250–440 mg per day. Thus, a biofuel cell in the configuration we propose consumes glucose at a rate of at most 2.8–28% of the rate at which cerebrospinal fluid glucose is replenished; by comparison, physiologic fluctuations in cerebrospinal fluid glucose can exceed 25%. So availability of glucose is not likely to be a limiting factor and glucose usage by a biofuel cell of the kind we describe here should not interfere with normal physiologic processes. (At low glucose utilization efficiencies a more accurate model of CSF bulk flow might be required before this model can be considered sufficient, as the fraction of the total glucose flux available to a fuel cell as a result of CSF bulk flow will depend on the precise location of the fuel cell within the subarachnoid space.).

#### Availability and use of oxygen in the cerebrospinal fluid

Typical oxygen partial pressures in human cerebrospinal fluid are 25–50 mmHg [61]. These levels correspond to 35–70 *µ*mol L^−1^ or 1.1–2.2 mg L^−1^ if, as in [62], the solubility coefficient 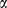 of cerebrospinal fluid is considered equal to that of plasma 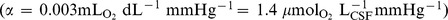. The bulk flow of oxygen through the subarachnoid space is therefore approximately 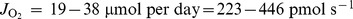. So physiologic oxygen levels are comparable to (slightly lower than) those under which biofuel cells such as those of Rabaey and colleagues have been tested. The electroreduction reactions of oxygen to water accompanying complete oxidation of glucose require 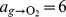 moles of oxygen per mole of glucose, so the oxygen consumption rate, 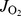, in the system we propose would be approximately
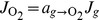
(7)

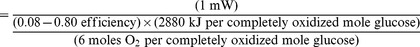
(8)

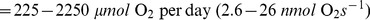
(9)


Thus, a biofuel cell in the configuration we propose consumes oxygen at a rate of 6–12 times the rate at which oxygen is replenished by cerebrospinal fluid bulk flow when operating at 80% efficiency, and ten times those rates when operating at 8% efficiency. Cerebrospinal fluid oxygen equilibrium under these conditions therefore depends on the ability of oxygen concentrations in cerebrospinal fluid and brain tissue interstitium to equilibrate through oxygen diffusion. A brain-implanted biofuel cell would exploit this equilibrating mechanism as a natural analog to continuous aeration of the cathode compartments of some laboratory-built biofuel cells. The diffusional transport characteristics of oxygen are such that equilibrium can be maintained with only a negligible perturbation to cerebrospinal fluid and interstitial oxygen concentrations, as the following calculations demonstrate.

As Lu and colleagues describe mathematically [62], the oxygen concentration *C* in the cerebrospinal fluid normally depends on four factors: (1) The oxygen partial pressure 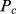 in the choroid plexus capillaries, whose content is filtered and transported through the choroid ependymal cells to become cerebrospinal fluid; (2) The oxygen partial pressure 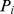 within the brain tissue interstitial fluid; (3) The formation rate 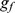 of cerebrospinal fluid; and (4) The drainage rate 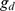 of cerebrospinal fluid. Under normal conditions 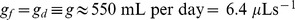. Oxygen partial pressures in blood plasma, interstitial fluid, and cerebrospinal fluid are proportional to oxygen concentrations through solubility coefficients that have approximately the same value for all three fluids: 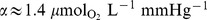. In the presence of a biofuel cell of the kind described here, the oxygen concentration also depends on a fifth factor: (5) The rate 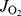 at which the biofuel cell consumes oxygen. The rate of change of the concentration of oxygen in cerebrospinal fluid can therefore be expressed by the following equation:
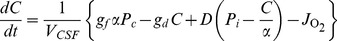
(10)where 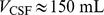 denotes the total volume of cerebrospinal fluid in the subarachnoid space, and 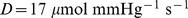 is the diffusion capacity of oxygen between the interstitial fluid and the cerebrospinal fluid [62]. The five terms within the curly braces can be understood as follows: (1) The concentration *C* increases as oxygen flows into the cerebrospinal fluid from the choroid plexus capillaries at a rate proportional to 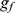 and concentration 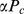, hence the term 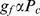; (2) The concentration *C* decreases as oxygen flows out of the cerebrospinal fluid at rate proportional to 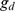 and concentration *C*, hence the term 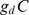; (3&4) Oxygen diffuses into the cerebrospinal fluid from the interstitial fluid in proportion to the partial pressure gradient between the interstitial fluid (at oxygen partial pressure 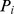) and the cerebrospinal fluid (at oxygen partial pressure 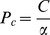), with a proportionality constant of *D*, the diffusion capacity; (5) Oxygen is consumed by the biofuel cell at rate 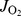. When the oxygen concentration *C* is at steady state, its rate of change vanishes, allowing us to solve for the equilibrium oxygen concentration, 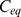:



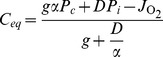
(11)

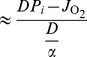
(12)


where the approximation holds because the diffusion transport is much faster than the bulk flow transport, allowing us to neglect the bulk flow terms containing *g* (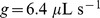 is six orders of magnitude smaller than 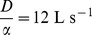; and since 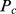 and 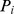 are on the same order of magnitude, 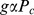 is six orders smaller than 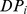). Equation 11 permits us to compare the equilibrium concentrations of oxygen in the cerebrospinal fluid in the presence (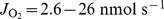) and absence 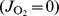 of the biofuel cell:
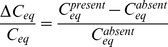
(13)

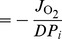
(14)

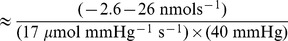
(15)

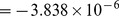
(16)


Thus, *the fractional change in oxygen concentration in the cerebrospinal fluid due to the presence of the biofuel cell will only be a few parts per million.* (The precise value of the interstitial pressure, approximated here as 40 mmHg, does not greatly impact this result). Note that although oxygen consumption by the biofuel cell will draw oxygen out of brain tissue into the cerebrospinal fluid, the corresponding decrease in interstitial fluid oxygen concentration will be even smaller in magnitude than the decrease in the cerebrospinal fluid, since the intracranial interstitial fluid volume is approximately twice that of the cerebrospinal fluid [62].

#### Oxygen equilibration time in cerebrospinal fluid

We can also estimate the time scale over which oxygen concentrations equilibrate in the cerebrospinal fluid. The system modeled by Equation 10 exhibits first-order kinetics with a time constant 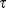 given by
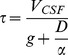
(17)

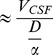
(18)

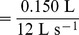
(19)

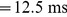
(20)


So the oxygen concentrations in this system equilibrate on the rapid timescale of tens of milliseconds.

#### Structure of oxygen concentration gradients in cerebrospinal fluid due to fuel cell oxygen uptake

To demonstrate that the surface-area-to-volume characteristics of our proposed brain-implanted biofuel cell are consistent with a functioning system, we can model the approximate structure of the concentration gradient field around the cathode. In a first-order approximation, the concentration of oxygen will approximately vanish at the surface of the cathode, where oxygen is electroreduced to water; and at a certain distance *d* (which we will determine) from the surface the concentration will be equal to *C*, the average oxygen concentration of the cerebrospinal fluid. If *d* is much smaller than the characteristic dimension 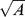 of the cathode (where *A* denotes the cathode area) and also much smaller than the width of the subarachnoid space at any location, the oxygen concentration 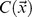 as a function of position will be approximately 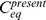 everywhere except within a region close to the cathode surface; within that region the concentration satisfies 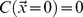 at the cathode surface, and rises to 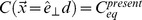 at a distance *d* normal to the cathode surface.

We can demonstrate that the diffusion characteristics of oxygen in the cerebrospinal fluid set *d* to a value consistent with efficient fuel cell operation in a brain-implanted system. We have demonstrated that the average concentration of oxygen in the cerebrospinal fluid equilibrates rapidly to 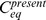 when the fuel cell is operating, so the spatial distribution 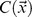 is effectively constant at times more than several 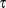 (several tens to hundreds of milliseconds) after the biofuel cell is turned on. We can therefore use Fick’s First Law of Diffusion to determine *d*:
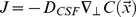
(21)

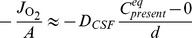
(22)where the first equation is a statement of Fick’s First Law for our system, and the second line expresses the first-order approximation we just described. The parameter 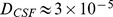 cm^2^ s^−1^ is the diffusion coefficient of oxygen in cerebrospinal fluid (approximately equal to that in water), 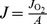 is the oxygen flux (here defined as an area-normalized flow) near the surface of the cathode, and 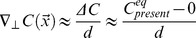 is the oxygen concentration gradient near the cathode surface (linearized in this first-order approximation). Therefore,
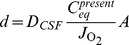
(23)so if the physiologic range of C is 35–70 μmol l−1, d can be at most 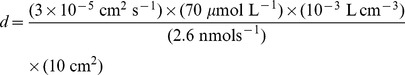
(24)

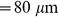
(25)for a device with surface area as great as *A* = 10 cm^2^. The value *d* = 80 μm satisfies our requirements that the concentration gradient vanish over a length scale much smaller than the typical dimension of the cathode and much smaller than the smallest width of the subarachnoid space, but it is also large enough to be physically achievable, as it is orders of magnitude larger than the mean free path of an oxygen molecule in water [63]. Thus, oxygen diffusion to the cathode in the system we propose will not be limited by the geometry of the system.
